# Exploring agricultural landscape change from the second half of the twentieth century onwards: combining aerial imagery with farmer perspectives

**DOI:** 10.1007/s10980-024-01914-z

**Published:** 2024-06-20

**Authors:** Franziska Mohr, Robert Pazur, Niels Debonne, Rebekka Dossche, Julian Helfenstein, Samuel Hepner, Christian Levers, Peter H. Verburg, Matthias Bürgi

**Affiliations:** 1 Swiss Federal Research Institute WSL, Zürcherstrasse 111, 8903 Birmensdorf, Switzerland; 2 Institute of Geography, University of Bern, Bern, Switzerland; 3 Institute of Geography, Slovak Academy of Sciences, Bratislava, Slovakia; 4 Environmental Geography Group, Institute for Environmental Studies (IVM), Vrije Universiteit Amsterdam, Amsterdam, The Netherlands; 5 Department of Antiquities, Philosophy, History (DAFIST),, Università Di Genova, Genoa, Italy; 6 Soil Geography and Landscape Group, Wageningen University, Wageningen, The Netherlands; 7 Agroecology and Environment, Agroscope, Zurich, Switzerland; 8 Thünen Institute of Biodiversity, Johann Heinrich Von Thünen Institute-Federal Research Institute for Rural Areas, Forestry, and Fisheries, Brunswick, Germany

**Keywords:** Agricultural landscape, Landscape history, Land-use change, Mixed-method approach, Object-based image analysis, Oral history

## Abstract

**Context:**

Anthropogenic landscape change is an important driver shaping our environment. Historical landscape analysis contributes to the monitoring and understanding of these change processes. Such analyses are often focused on specific spatial scales and single research methods, thus covering only limited aspects of landscape change.

**Objectives:**

Here, we aim to assess the potential of combining the analysis of historical aerial imagery and local stakeholder interviews for landscape change studies using a standardized mapping and interviewing approach.

**Methods:**

We compared six agricultural landscapes across Europe and mapped land-cover using historical aerial imagery (starting between 1930 and 1980, depending on data availability, until recent years) with an object-based image analysis and random forest classification. For local perspectives of landscape change, we conducted oral history interviews (OHIs) with (almost) retired farmers. Comparing recorded landscape changes from both approaches provided insight into advantages of combining these two methods.

**Results:**

Object-based analysis enabled the identification of high-resolution land-cover dynamics, with scale enlargement and cropland/grassland expansion being the most commonly recurring trends across European landscapes. Perceived landscape changes identified in the OHIs included changes in farm management, landscape structure, and infrastructure. Farmers also reported drivers and personal values associated with landscape change. Combining the two historical landscape analysis tools resulted in a qualitative and quantitative understanding of changes in land-cover, land use, and land management.

**Conclusions:**

Comparing physical land-cover change with local farmer perspectives is key to a comprehensive understanding of landscape change. There are different ways the two methods can be combined, leading to different venues for science and policy making.

**Supplementary Information:**

The online version contains supplementary material available at 10.1007/s10980-024-01914-z.

## Introduction

Landscape change influences biodiversity and ecosystem services (Tscharntke et al. 2005; van Zanten et al. 2014), but also heritage and the social and personal identity of local actors (Dossche et al. 2016; Arnaiz-Schmitz et al. 2018). In Europe, most of the land area is managed and thus directly shaped by human actions (Antrop 2004). It is critical to manage these lands in more sustainable ways, as unsustainable land use and management are detrimental to ecosystem functioning (Helfenstein et al. 2020). As landscape is an important action field for more sustainable land use (Weltin et al. 2018), understanding landscape change is essential (Bürgi et al. 2022).

The study of land-use and land-cover change (LULCC) and the proximate and underlying drivers thereof (García-Martín et al. 2021) has become a focus of landscape change research. Complementary to this aspect of landscape change, there has also been a growing interest in landscape perception, reflected in the European Landscape Convention, which emphasizes landscape as “an area as perceived by people” (Council of Europe 2000). Research based on this topic typically examines landscape change through the lens of, e.g. landscape identity (Butler and Sarlöv-Herlin 2019) or planning (Hersperger et al. 2020; Gonçalves and Pinho 2023). In recent years, landscape change research progressed through an increase in the efficiency of land-cover mapping (Sertel et al. 2022; Feizizadeh et al. 2023), as well as an improved understanding of perceived landscape changes based on participatory approaches (Lubis and Langston 2015; Fagerholm et al. 2021).

Spatial data is a critical source for landscape change research, and improved availability of spatial data in recent years has opened new doors for studying and understanding landscape change. Specifically, advances in digitization and open data policies have increased the accessibility and availability of historical digital maps and aerial imagery. Comparing historical and recent topographical maps, e.g. through manual digitization, can provide insights into long-term historical landscape changes (Loran et al. 2018; Matasov et al. 2019). Meanwhile, recent advances in machine learning and computational capabilities have enabled progress in the automated identification of landscape structures in historical maps, including buildings (Heitzler and Hurni 2020), roads (Jiao et al. 2024), and sand dunes (Groom et al. 2021), reducing the effort of manually digitizing such maps. However, maps are an abstraction of reality, and landscape representation in maps is strongly dependent on the mapping method and focus issued by the responsible authority. Further, the definitions of map elements can differ between mapping products (Svenningsen et al. 2022). For example, the definition of wetlands in Swiss maps changed from ‘not passable by horse’ in earlier maps toward a distinction based on reed vegetation (Gimmi et al. 2011). The semantic discrepancies between maps for different regions or points in time hence limit their comparability.

Historical aerial imagery and satellite imagery provide an additional important source of information regarding past landscape change, although mostly restricted to the second half of the twentieth century (Baessler and Klotz 2006; Brown et al. 2018; Wang et al. 2022). While historical aerial imagery has been used to manually digitize land use and assess its change (Baessler and Klotz 2006), there has been a recent increase in studies using automated classification of historical aerial imagery, based on both pixel-based approaches (Ratajczak et al. 2019) and object-based image analysis (OBIA) (Dimopoulos and Kizos 2020; Kindermann et al. 2023). The (semi-)automated analysis of (historical) aerial imagery enables the processing of larger areas, more study sites, and more time steps (Ratajczak et al. 2019). The advantages of historical aerial imagery over satellite imagery are its larger time lapse and its corresponding finer spatial resolution (e.g., 1 m). In many European countries, historical aerial imagery is available from as early as the 1950s, or sometimes even the 1920s, while the first satellite imagery dates back to the 1960s (Corona spy satellite imagery; Nita et al. 2018) or to 1972 (first Landsat generation). The finer spatial resolution of the aerial imagery enables the detection of land-cover classes, but also changes in landscape structures that are important for landscape-scale analysis (Baessler and Klotz 2006; Skokanová et al. 2020; Helfenstein et al. 2024). This latter capability was not possible using standard satellite imagery such as Landsat.

While spatial analysis based on aerial imagery for specific target years can be a valuable tool for detecting landscape change, the results are often limited in thematic detail and can hence easily lead to misinterpretations (Breidenbach et al. 2022). Including complementary data, for example derived from oral history interviews (OHIs) or other participatory methods, can help overcome the limitation of LULCC identified solely from remote sensing methods (Bürgi et al. 2017; Dimopoulos et al. 2023), and there is a great diversity in how spatial analysis can be combined with participatory methods. While stakeholder perception can be directly compared with landscape-change maps (Malek et al. 2014; Fox et al. 2017), it can also be used to inform land-cover classification based on remote sensing data (Isager and Broge 2007; Berget et al. 2021). Stakeholder perspectives can further provide insights into potential driving forces of landscape change (Malek et al. 2014; Fox et al. 2017; Dimopoulos and Kizos 2020). OHIs make it possible to assess long-term landscape changes from actors’ perspectives (Bürgi et al. 2017; Mohr et al. 2023a). This method originated in the historical sciences, where it is often used to supplement insights from written sources by giving voice to traditionally unrecorded groups of actors (Schaffner 2013; Abrams 2016), and is now used in a variety of disciplines.

Here, our overarching goal is to determine how long-term landscape change across different agricultural landscapes in Europe can be better understood by combining different methods. We therefore leverage the advantages of mapping landscape change based on historical aerial imagery and perceived landscape changes from OHIs with local farmers in a mixed-methods approach. Our data covers the period from 1931 to today (depending on study site) and the following six regions in Europe: Santa María del Páramo in Spain, Ille-et-Vilaine in France, Reusstal in Switzerland, Flevopolder in the Netherlands, Querfurter Platte in Germany, and Turzovka in Slovakia. By analyzing multiple study sites, we aim to identify diverse landscape change pathways and underline the complementarity of the results of the two approaches. Specifically, we ask the following questions:

- Which landscape changes are detected using remote sensing on historical aerial imagery?
- What kind of landscape changes are reported in OHIs?
- How do insights from these different approaches complement each other?

## Methods

To analyze landscape changes in our six study sites (see "Study sites"), we applied a mixed-methods approach. We mapped long-term historical land-use change using a time series of aerial images and a supervised classification based on machine learning (see "Remote sensing"). We conducted OHIs in all study sites and used the resulting transcripts to gain a bottom–up perspective on perceived landscape changes by farmers (see "Oral history interviews" section). The two methods were chosen as complementary source types, each informing about different aspects of the landscape. After the separate analysis, the results from the two methods were compared in a concurrent triangulation approach (Creswell 2003).

### Study sites

**Fig. 1:**
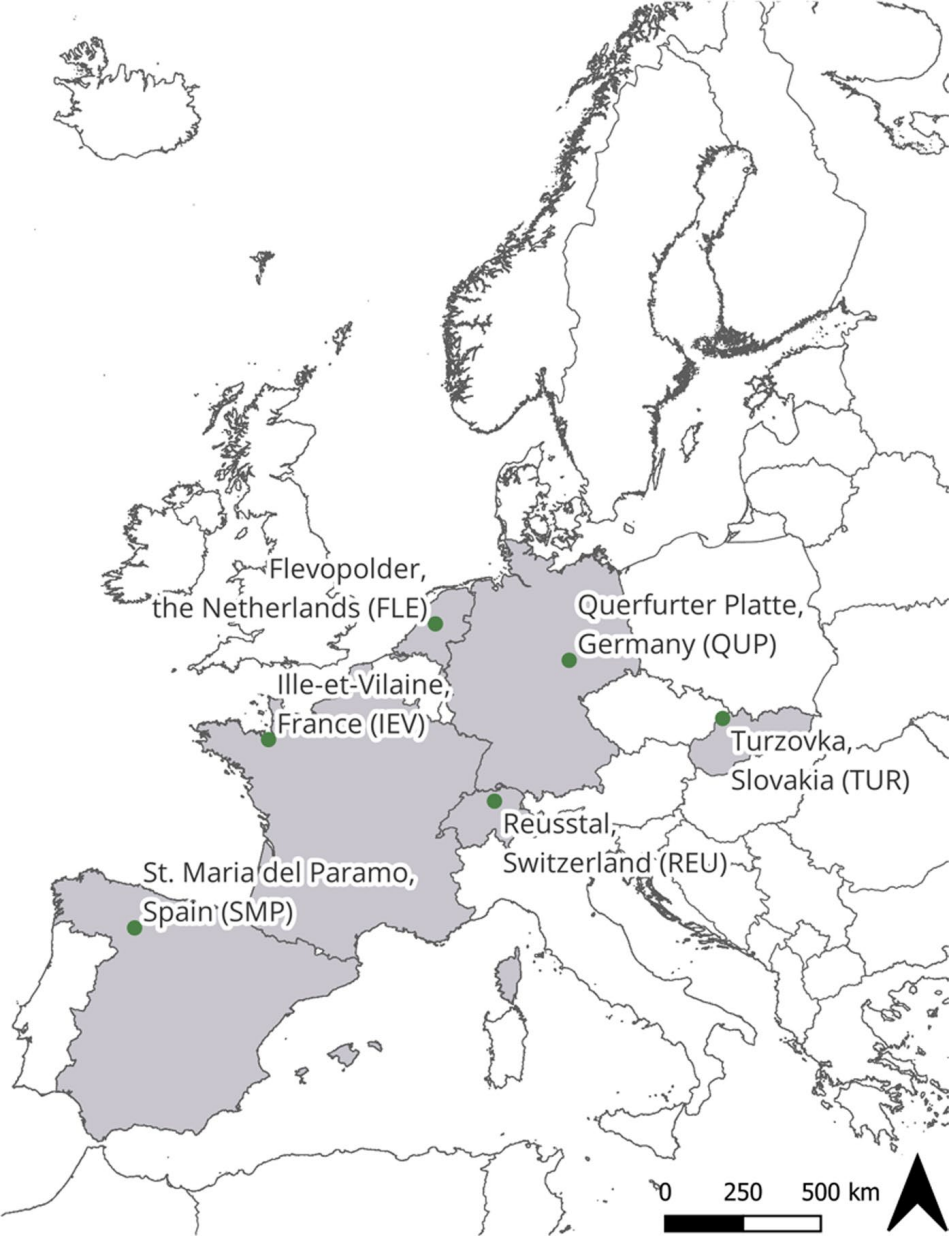
Location of the six study sites. See Table 1 for additional information

To go beyond insights from a single case study, we opted for a comparative research approach. We selected six study sites of ~ 25 km^2^ (Fig. 1; Table 1) from a set of study sites presented in Diogo et al. (2023), with the aim of covering a variety of agricultural landscapes, farming systems, and biophysical conditions (Helfenstein et al. 2024; Mohr et al. 2023b). The selected study sites have a good data availability and share a focus on arable or mixed farming systems. In Santa María del Páramo (SMP), Flevopolder (FLE), and Querfurter Platte (QUP), landscapes are dominated by highly intensive arable agriculture, with some farms in QUP also using their crops for large-scale livestock production. The landscapes of Ille-et-Vilaine (IEV), Reusstal (REU), and Turzovka (TUR) are more diverse, with arable land, grassland and forest, and farms often focus on livestock production. A recent study showed that, while farms in FLE and QUP have increased in multifunctionality over the last 20 years, SMP, IEV, and REU are still on a productivist pathway and TUR is trending toward marginalization (Helfenstein et al. 2024). FLE is located on an artificial land reclamation that was built in the 1950s, mainly for agricultural purposes. QUP and TUR both experienced a period of centrally planned economy during socialism, from the end of World War II until 1989.

**Table 1:** Overview over the study sites considered

Study site abbreviation	Region	Country	Agricultural system	Source of aerial images^©^	Number of oral history interviews
SMP	Santa María del Páramo	Spain	Mainly arable cultures (+ few livestock farms)	Instituto Geográfico Nacional de España	10
FLE	Flevoland	The Netherlands	Mainly arable cultures (+ few livestock farms)	Dutch National Spatial Data Infrastructure (PDOK) (1980; 2020); Eurosense (2000)	10
QUP	Querfurter Platte	Germany	Mainly arable cultures (+ mixed farms with megastables)	GeoBasis-DE/LvermGeo ST	11
IEV	Ille-et-Vilaine	France	Mixed farms (dairy/suckler cow; arable cultures and pasture)	Institut national de l’information géographique et forestière	9
REU	Reusstal	Switzerland	Mixed farms (dairy/suckler cow; pigs, arable cultures & pasture)	Swisstopo	10
TUR	Turzovka	Slovakia	Livestock (+ mainly pasture)	Geodesy, Cartography and Cadastre Authority of the Slovak Republic	7

**Fig. 2:**
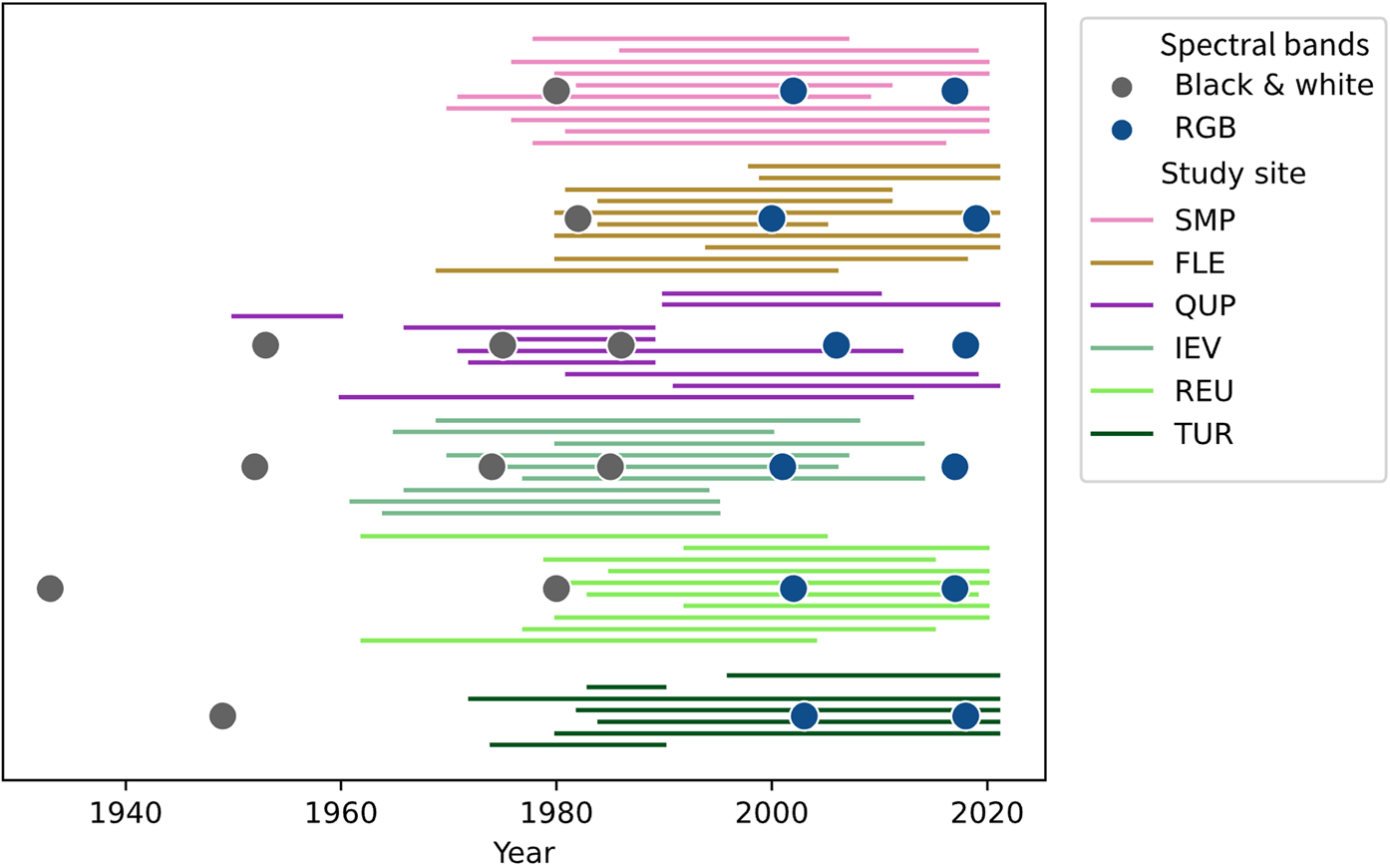
Active working times (lines) of the farmers interviewed in oral history interviews (OHIs), used as a proxy for the period covered by the OHIs, and the years for which aerial images were available (circles). The colors of the circles indicate whether the aerial image used for our remote-sensing-based landscape-change analysis was recorded in black and white (BW) or color (RGB). See Table 1 for full names of study sites

Depending on the availability of data for the study sites, LULC mapping started between the 1930s and the 1980s and covered ~ 20-year intervals until recent years, while the period covered by the OHIs interviews goes back to the 1960s when the oldest interviewees started to farm (Fig. 2).

### Remote sensing

We obtained aerial images from national archives (Table 1). The images varied in spatial resolution and quality (e.g., different types of image noise and color balance), and covered periods from 1931 to 2020 (Fig. 2).

### Segmentation of the aerial imagery

For the segmentation, we pre-processed the images to limit the level of grain noise by applying a pretrained model based on deep latent space translation, originally developed to restore old photos (Wan et al. 2020). We used the pretrained model with a default set of parameters and visually inspected the results before segmentation.

We segmented the aerial images to identify homogeneous land-cover patches and landscape structures using a combination of two segmentation approaches. For the edge detection, we parameterized a pre-trained convolutional neural network model. We tested different edge detection algorithms and selected a dense extreme inception network, which was found to be more robust compared with other state-of-the art edge detection algorithms by Soria et al. 2023. For patch delineation, we used a pre-trained robust segmentation model that identified unique features within the landscapes, such as single fields and forest patches. We applied the Segment Anything model (ViT-H SAM), a deep learning model, which has been built over 1 billion objects delineated on over 11 million images (Kirillov et al. 2023).

Applying the edge detection and segmentation model over the aerial photos resulted in grayscale images highlighting the borders between different landscape features. These grayscale images were subsequently converted into binary images and vectorized to form spatial polygon datasets. Individual derived segments were interpreted as unique landscape features. When inspecting the resulting polygons visually, we observed that there were polygons that collapsed large areas because the border between landscape elements was not captured adequately. We thus selected—apart from in QUP—all polygons larger than 30 ha and used a more sensitive delineation of the two segmentation models. However, some unrealistically large and branched polygons remained, which we excluded in the pre-processing steps for the classification.

### Classification

As the pre-processing of the aerial imagery chosen for the segmentation had an impact on the texture of the land-cover classes, we decided on a different pre-processing step of the aerial imagery for the classification (see Fig. 1 in Supplementary Information (SI) I for an overview of the classification approach). To address the variation in the quality of the aerial imagery, resulting from differences in spatial resolution (between 0.1 m^2^ and 1 m^2^) and spectral resolution (black and white [BW] or color [RGB]) across the study sites and years, we synchronized the datasets by transforming all RGB images to BW (Kanan and Cottrell 2012) and resampling all images to 1 m^2^ pixel size. We further slightly smoothed the images using a Gaussian blur (sigma = 2, window size = 5).

To classify land-cover and compare it across time steps, we developed a land-cover nomenclature (Table 2) that ensured that all classes were identifiable in all time steps. Using the nomenclature, we manually labeled polygons from the segmentation for each land-cover class, time step, and study site, resulting in a total of 1088 polygons to be used as training/testing data. To create a robust model, we only labeled polygons with one land-cover class for the training/testing data.

**Table 2:** Nomenclature used for land-cover classification

Class	Description	Visual characteristics
Built-up	All types of artificial surfaces, including settlements, roads and railways, and industrial areas	Polygons of small (single parts of houses) compact areas or large incompact (road networks) areas. Often characterized by very light and homogeneous gray shades
Cropland	Cropland, including rotational grassland	Cropland could have different shades of gray, ranging from light gray (usual) to dark gray (e.g., corn), depending on crop type and the timing of when the aerial image was taken. This land-cover class sometimes contained linear rows (tractor tracks, planted rows), but in cases of lower resolution these appeared as homogeneous areas Typically, the parcels of arable land had a rectangular shape and larger area
Grassland	Pastures, meadows, and other sorts of grassland areas, such as field and street margin vegetation	Open, vegetated non-forested areas with less geometrical shapes than arable land. The texture is coarse, with no directional patterns. Mowing tracks often showed circular patterns
Forest/trees	Forests, lines of trees and single trees	Dark gray shades and often rough surfaces caused by the tree tops (compared with the other land-cover classes) Area sizes ranged from very small to very large
Water	Water courses and water bodies; not prevalent in most study areas	Smooth and homogenous surfaces. Gray shades vary from very light to very dark, depending on the water surface reflectivity (position from which the image was taken) and water turbidity

For all polygons, we defined a set of predictors that describe the spectral characteristics and geometry of the land-cover classes, allowing separation of specific land-cover classes from others within the nomenclature. To optimize the models, we tested the variability of the predictors and validated them based on their explanatory power, their correlation to other predictors, and their partial dependence on the different land-cover classes. The final set of predictors (Table 1 in SI I) included gray intensity, area, shape index (iso-perimetric quotient to describe the compactness of the polygon; e.g., Hernandez-Suarez et al. 2022), and texture measures (contrast, correlation, entropy) based on the gray level co-occurrence matrix (GLCM; Hall-Beyer 2017). As the pixels close to the polygon border often depicted a mixture of land-cover classes (i.e., edge effect), we shrank polygons larger than 10 m^2^ by 0.5 m [unless this yielded an invalid result (NA)] to calculate the gray intensity and GLCM metrics for each polygon.

As the classification model, we used a random forest classifier (Breiman 2001) available in the *scikit-learn* package (version 1.4.0) in Python 3.9, as it has shown good performance for LULC mapping (Modica et al. 2021; Adugna et al. 2022). To train the model, we set the number of decision trees to 100 and used 80% of the labeled data for training and 20% for testing. We excluded polygons with NA values for any of the predictor variables. We built two models based on groups of similar landscapes: one for arable landscapes (MA; for study sites SMP, FLE and QUP) and one for mixed landscapes (MM; for study sites IEV, REU and TUR). We used the same set of predictor variables for both models. We used the labeled data from the arable landscapes to train/test MA and the labeled data from the mixed landscapes to train/test MM. To assess model accuracy, we calculated overall accuracy, out-of-bag score, Cohen’s kappa coefficient, and producer/user accuracy.

**Fig. 3:**
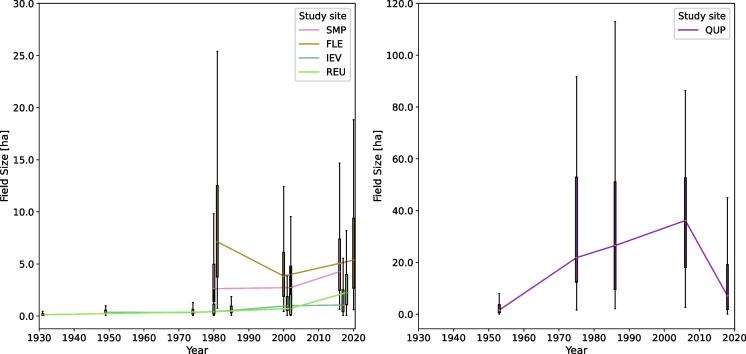
Evolution of median field size for all study sites except TUR. Polygons classified as crop smaller than 500 m^2^ were excluded. To improve interpretability, the date of the last aerial photograph in SMP and REU was shifted by minus/plus 1 year. Because the field size for QUP is significantly larger than that for the other study sites, it is plotted separately with a different y-axis. As TUR focused mainly on grassland management after 1949, we excluded it from this analysis

Based on the classification results, we decided to compare the size of arable fields across study sites and time steps. For this, we calculated the median area of all polygons of the cropland type that were larger than 500 m^2^ per study site and time step (Fig. 3). We further evaluated the land-cover classification for all time steps/study sites based on visual comparison with the corresponding aerial image. To exclude land-cover changes that were clearly due to misclassification, we corrected the classification of patches that showed persistence for the beginning and end, but not for any time step in between (e.g., a sequence built-up—cropland—built-up would be corrected using this approach; see Table 2 in SI I for a list of corrections). From the results, we then summarized the land-cover transitions and persistence, which are visualized in Fig. 6.

### Oral history interviews

#### Interview procedure and questionnaire

To obtain a long-term stakeholder perspective on agricultural and landscape change, we conducted between 7 and 11 OHIs per study site (Table 1), with experienced farmers who were either retired or nearing retirement (Fig. 2). We focused on elderly farmers for two reasons: Given that the landscapes studied are dominated by agricultural land use, we were interested in gaining insight into the perspectives of farmers who, in their role as landscape managers, are co-responsible for some of the long-term changes (Primdahl et al. 2013). They are also directly affected by landscape change in their working and living environments. Second, focusing on one stakeholder group increased the homogeneity of the sample, which allowed us to plan for 10 interviews per study site (Robinson 2014), which in turn permitted the consideration of multiple study sites.

We used a questionnaire translated into the local language of the study sites, with open-ended questions in the first part, including a question on landscape change. The second part consisted of semi-structured questions on different aspects of on-farm changes. Here, we focused solely on the responses related to the question on landscape change. Because the interview design allowed a relatively open conversation, there were additional interactions between the interviewee and interviewer beyond the predetermined questions. We considered statements from other parts of the OHIs as context information if clearly linked to the topic. For conducting the OHIs, we relied on a network of local academic study partners. To find interviewees, we used a snowball sampling strategy based on pre-existing contacts, door-to-door interviews, or recommendations by either local associations or farmers. We conducted and recorded all OHIs in the local language, then transcribed the recordings and translated them into English. A detailed description of the interview procedure and questionnaire is provided by Mohr et al. (2023b). Prior informed consent was obtained from all interviewees, and the experimental design and the questionnaire received ethical clearance from the Ethical Commission of the Swiss Federal Institute of Technology (ETH-EK 2020-N-146), and, where required, from the relevant authorities in the participating countries.

#### Analysis of oral history interviews

Following qualitative content analysis (Erlingsson and Brysiewicz 2017), we analyzed the interview data for mentioned landscape changes and inductively created categories for each identified change type, such as ‘increase in field size’ or ‘conversion from grassland to cropland’. Based on these categories, we defined seven overarching themes of landscape change (see Table 1 in SI II for a list of themes and categories). We then calculated the frequency with which each category was mentioned per study site. We counted each category only once per interviewee. Because the number of OHIs varied between study sites (Table 1), we normalized absolute numbers to percentages to allow comparisons between study sites. We complemented our analysis with narratives for each study site, relating values and drivers to perceived landscape changes (SI II).

## Results

### Mapping landscape change using aerial imagery

We found that image segmentation delineated most landscape features correctly. However, the segmentation tended to omit elements with low gray intensities, especially prevalent in aerial images with low quality. It also tended to overestimate structures within land-cover patches, e.g., for tree tops in a forest or driving tracks within a crop field. Alternation of land-covers within a smaller area (e.g., transition from field border to hedgerow to field margin vegetation to road) and complex and diversified landscapes (in study sites IEV and REU) were especially prone to such inaccuracies.

Our land-cover models reached an overall accuracy of 0.89 and 0.86 for arable landscapes (MA) and mixed landscapes (MM), respectively. MA performed slightly better than MM for Cohen’s kappa and out-of-bag score (Table 3). Both models varied slightly in terms of producer’s/user’s accuracy, as also shown in the confusion matrix (Fig. 2, SI I). While for MA most misclassifications occurred between grassland and forest/trees, for MM grassland was often misclassified into one of the other classes, especially cropland. We also observed mismatches between built-up area and cropland.

**Table 3:** Overview over the metrics calculated to assess the performance of the trained random forest model on the test data

Measure	Scores for model trained on arable landscapes (MA)	Scores for model trained on mixed landscapes (MM)
Overall accuracy	0.89	0.86
Cohen’s kappa	0.85	0.81
Out-of-bag score	0.87	0.81
Producer’s accuracy	Built-up: 0.93 Cropland: 0.96 Grassland: 0.80 Forest/trees: 0.80 Water: 1	Built-up: 0.89 Cropland: 0.95 Grassland: 0.74 Forest/trees: 0.9 Water: 1
User’s accuracy	Built-up: 0.93 Cropland: 0.98 Grassland: 0.52 Forest/trees: 0.95 Water: 0.67	Built-up: 0.93 Cropland: 0.70 Grassland: 0.9 Forest/trees: 0.94 Water: 0.60

We observed a general increase in the median field size of cropland over time, especially in the last decades (Fig. 3). In terms of field size, there are considerable differences between the study sites, with QUP having significantly larger field sizes than anywhere else, and the arable landscapes (FLE, SMP) having larger field sizes than the mixed landscapes (IEV, REU). While the trend for SMP, IEV and REU is more or less within the range expected from visual comparison with the respective aerial images, FLE shows a steep decrease between 1981 and 2000 as a result of subdivisions that were not mapped in 1981. A special case is QUP, where the median field size increased strongly between 1953 and 1975 subsequent decrease from 2010 to 2018. Based on a visual assessment, uncertainties in field sizes are due to improperly derived polygons (collapsing too many units/describing too much detail) and to a lesser extent to misclassifications, so absolute values should be used with caution while overall trends are mostly reliable. See Table 1 for full names of study sites.

**Fig. 4:**
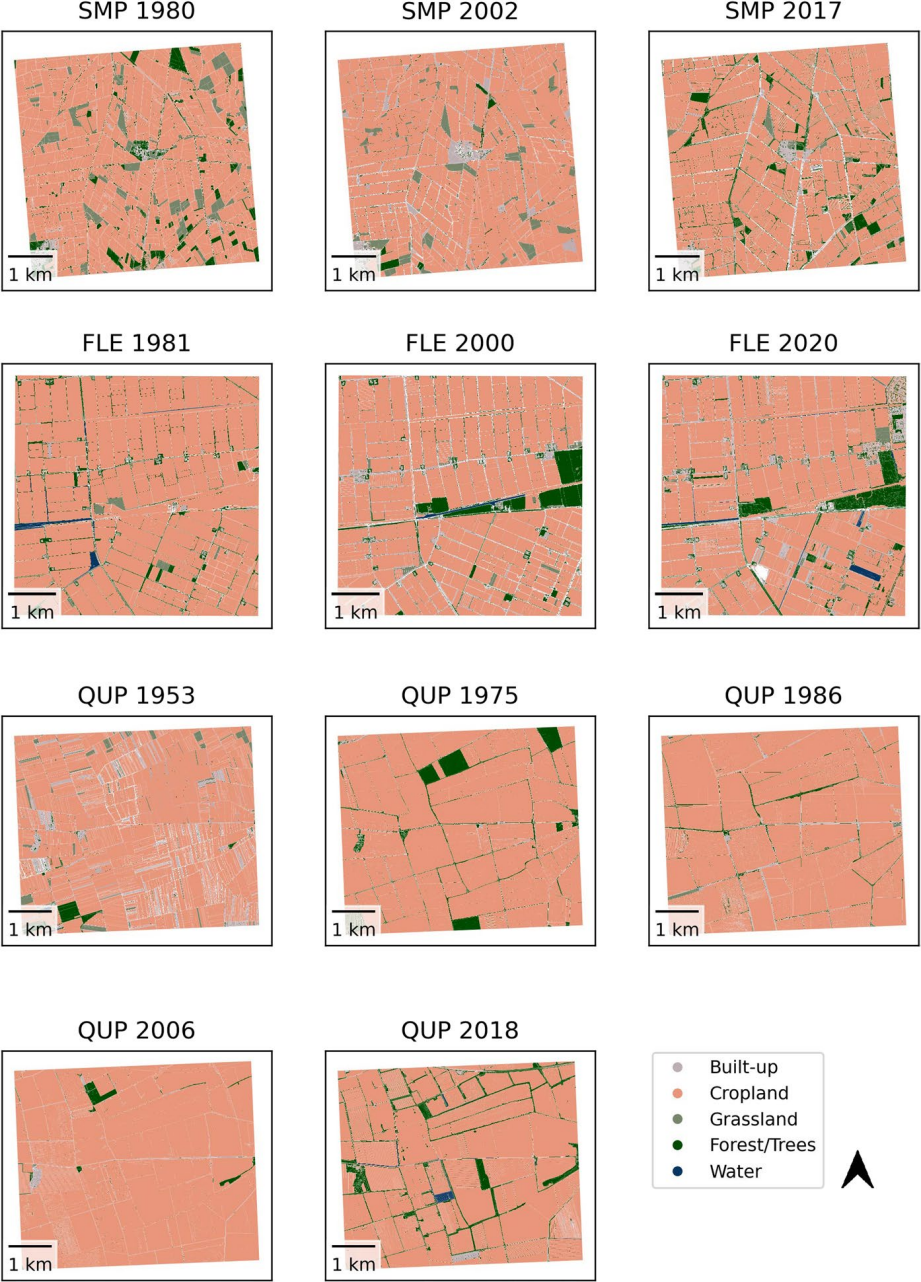
Mapped land-cover for study sites in arable landscapes. See Table 1 for full names of study sites

**Fig. 5:**
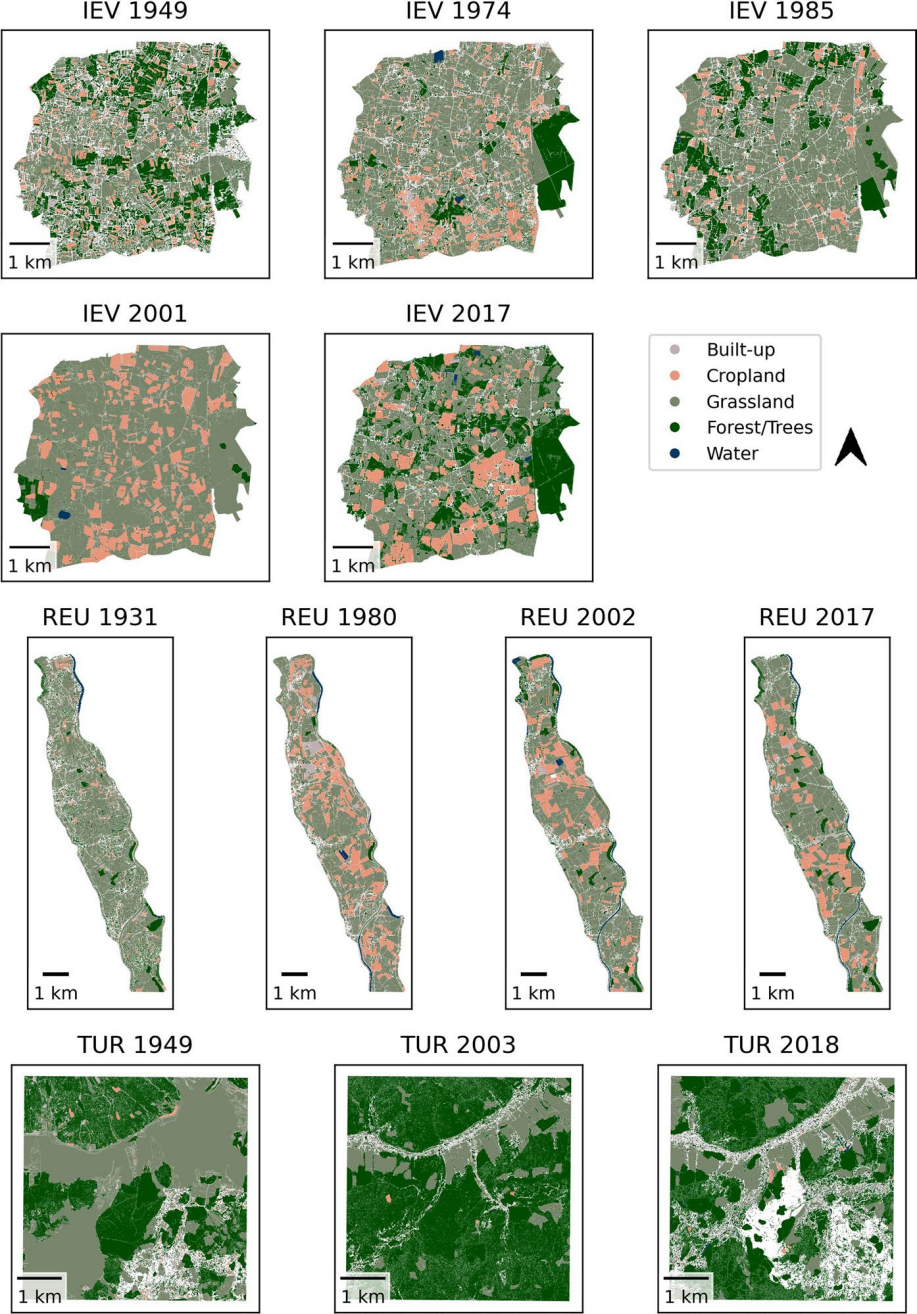
Mapped land-cover for study sites in mixed landscapes. See Table 1 for full names of study sites

**Fig. 6:**
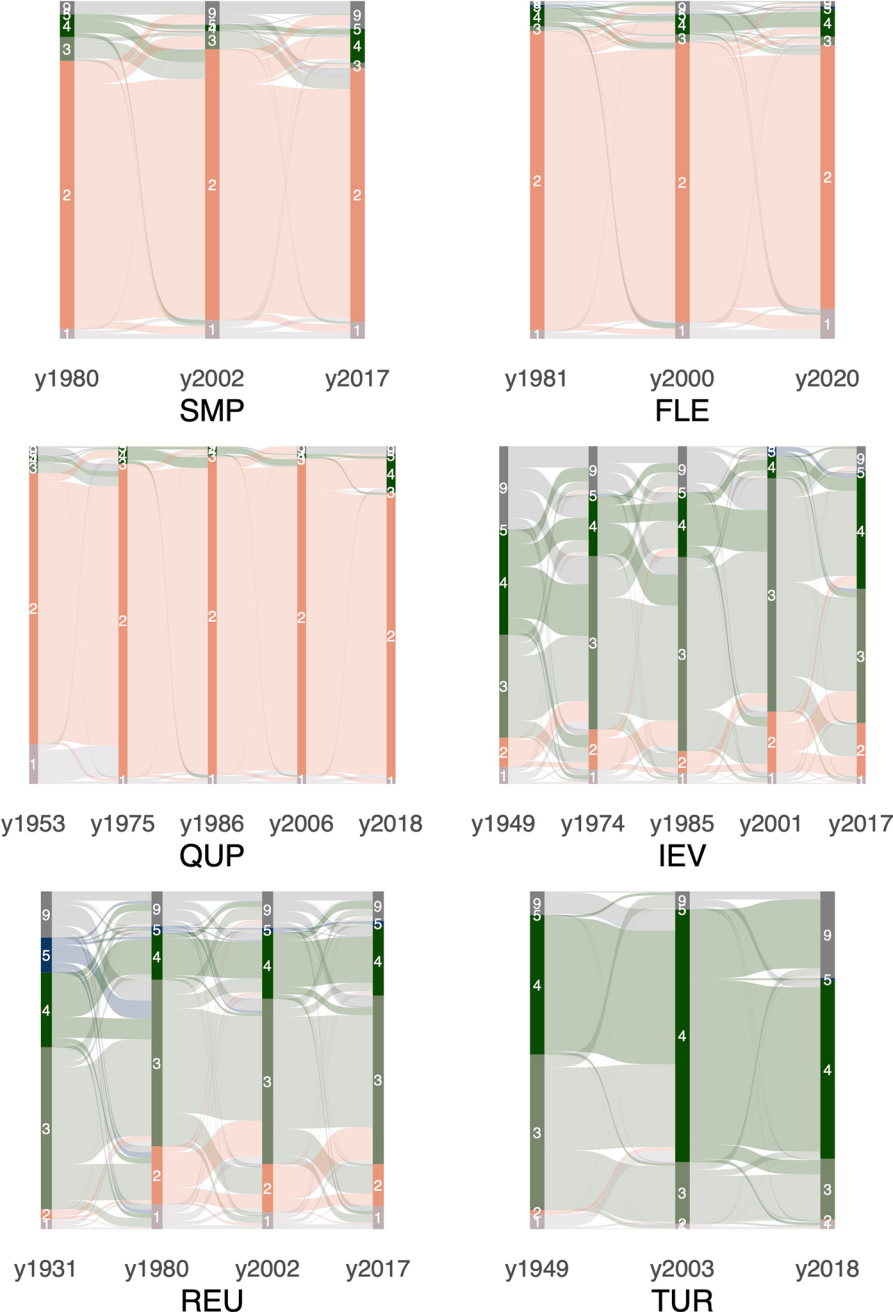
Landcover persistence and transitions in the different study sites. The numbers correspond to following land-cover classes: 1 = built-up, 2 = cropland, 3 = grassland, 4 = forest/trees, 5 = water, 9 = N/A

The following landscape observations were made for the study sites based on the classified land-cover maps (Figs. 4, 5), the calculated total land-cover changes per study site (Fig. 6), and the original aerial imagery as a reference.

SMP is dominated by large fields that were partly misclassified as grasslands/built-up area/forest. Misclassified fields were often geometries that collapsed multiple fields together. Both the field structure depicted in the image and the average field size (Fig. 3) show an increase in parcel size for both time steps, but with a larger increase between 2002 and 2017. Overall, there is a persistence in cropland (Fig. 6).

FLE is characterized by structured and regular fields, regularly dispersed farms (Fig. 4) and persistence in cropland (Fig. 6). The forested area in the center-east of the study site was (partly) misclassified, especially in 1981. Occasionally, cropland with a dark gray shade was classified as water. In the northeast, the growing city limits of Dronten were captured in the year 2020, also visible in Fig. 6. As seen in Fig. 3, field sizes increased between 2000 and 2020; however, the mapped fields were larger in 1981. Based on visual inspection, we found that the algorithm was sometimes unable to identify and differ between multiple crops in the same parcel due to the high similarity of gray shades and the overall image quality, resulting in collapsed fields.

While QUP was dominated by arable agriculture for all five time steps (Figs. 4, 6), there was a remarkable increase in field size and structure between 1953 and 1975, both visible in Figs. 3 and 4. Between 2006 and 2018 there was a decrease in field size. Some fields were misclassified, mostly as forest. Especially in more recent images, structures in cropland, e.g., driving tracks, were segmented but then classified as forest/built-up. In the aerial images from 2006 and 2018 there are also visible windmills, classified mostly as built-up. While there was no highway visible in the study area during the socialist period, in 2006 the first structures were visible in the northeast (classified as arable land), and in 2018 the highway was built (classified as partly built-up/partly grassland). While the crop fields were classified relatively well, there were some problems with the other classes, e.g. geometries delineating roads (built-up) often also covered field edges and hedgerows, leading to varying classification across time steps.

IEV is a highly mosaiced landscape that challenged both the segmentation and the classification due to its magnitude of details. A comparison of the land-cover maps reveals a certain –tentative – increase in cropland over time (Figs. 5, 6) and, more clearly, an increase in plot size (Fig. 3). However, there is a large forest in the eastern part of the study area, which is inhomogeneous in structure and gray shade (the latter is true especially in the older imagery), resulting in not all parts being classified as forest; see IEV 2017 as a reference for the expected extent of the forest and Fig. 6 for irregular variability of the forest cover.

An increase in field structure and size is particularly evident for REU between 1931 and 1980. However, when specific regions are considered, such as the northernmost croplands, there is a further increase in size after 1980. Although the exact distribution of cropland and grassland is affected by segmentation and classification errors (e.g., geometries classified as built-up areas instead of cropland in 1980), the increasing importance of cropland between 1931 and 1980 is remarkable (Figs. 5, 6). The dispersed small forest/tree geometries, mostly within grassland, that were visible in 1931 were not as abundant thereafter. In addition, the settlement area of the villages scattered throughout the study area increases throughout the time series (Fig. 6, the high 1980 value is related to the misclassification mentioned above).

In TUR, small crop strips were visible in 1949, mainly in the main valley (upper half of image), but this cropland was converted to grasslands until 2003. Also, the forest grew toward the main valley from 1949 to 2003, (Figs. 5, 6) although this change was not as visible, due to a large forest geometry that also covers a substantial area of grassland. From 2003 to 2018 there is a massive clear-cut in the south-eastern quadrant of the image (partly not classified, but still tentatively visible in Fig. 6).

### Perceived and remembered landscape change

**Fig. 7:**
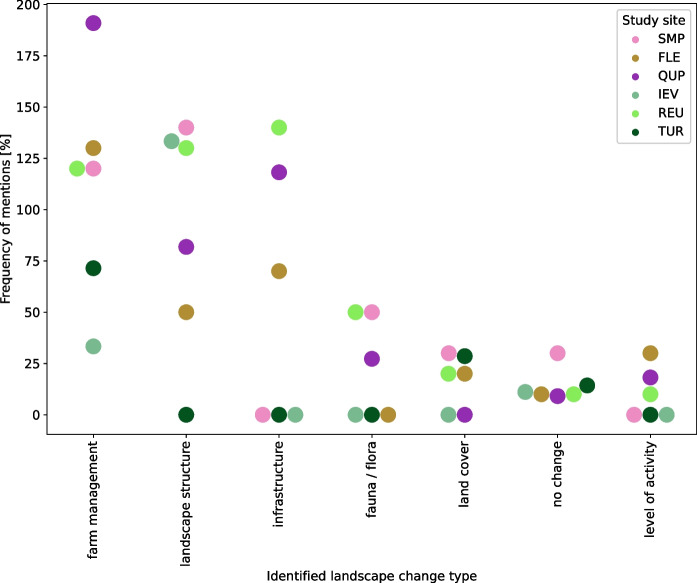
Landscape change types across the study sites. The landscape change types on the x-axis are ranked according to the overall prevalence with which they occurred across all study sites (left = high, right = low). Frequencies were calculated for each study site and category (Table 1, SI II) and then summed for the corresponding landscape change type. This means that the frequencies shown in the figure can exceed 100%. See Table 1 for full names of study sites

Farmers described a variety of landscape changes, with changes in farm management, landscape structure, and infrastructure frequently mentioned across all study sites, accompanied by changes in fauna and flora, land-cover, and level of activity that were perceived in selected study sites (Fig. 7; see SI II for details on the categories (Table 1) and study sites (Fig. 1). In each study site there were also a couple of interviewees who reported that they have not perceived any or only little landscape changes.

Changes in farm management were the most frequently reported landscape change and the only landscape change identified across all study sites. The most prevalent type of farm management change was related to increasing field size, but also to changes in farm strategy (e.g., changes in crops/livestock, adopting organic labels), landscape interventions (e.g., leveling of land, irrigation), and shifts in farm composition (e.g., trend toward larger and fewer farms, abandonment). Some interviewees reported partial greening due to private ecological initiatives (e.g., flower meadows, greener field margins). In a few interviews, the homogeneous maintenance of e.g., roadside ditches and railway embankments was criticized. Changes in landscape structure were often related to a decrease in field trees (often fruit trees) and formed an important part of the narratives in SMP, IEV and REU: “When we took over the land, while your grandfather was alive, one left the apple trees. But ten days after he died, we cut it all down. Because before, he didn’t want to, eh, ohlala. One shouldn’t have touched it. Because with the tractors around the apple trees, it was a mess, it wasn’t possible” (IEV, F7). Whereas in some sites a decrease in linear green elements, such as hedgerows or lines of trees was reported, especially for the period 1960–1980, other interviewees perceived an increase in both trees and linear green elements in more recent years. Infrastructural change was often related to the built environment, such as new highways/roads, train lines, and settlement expansions, but also to blue or green infrastructure, e.g. a newly founded conservation area in REU. Interviewees also reported changes in fauna and flora, such as decreases in the number of hares, frogs and plants, or increases in the number of wild boars and beavers. Observations related to land-cover included references to the persistence of cropland and the transformation into cropland, but also a change from arable land to forest in TUR. Some interviewees also reported ‘invisible’ changes in the landscape, for example the increasing noise pollution of wind turbines, not knowing the people of the village anymore, or increased activity due to the presence of more people and traffic in the landscape.

To illustrate perceived landscape changes (Fig. 7) in the study sites in more depth, we constructed narratives based on the OHI data (see SI II; Table 4 for a short version). These narratives include linkages between landscape change types, drivers and values attributed to perceived changes.

**Table 4:** Overview of typical landscape changes and related remarks in the study sites

Study site^1^	Key points from narratives (SI II)
SMP	- Intensification accelerated through development of large-scale irrigation systems and linked to land consolidation - Increased focus on cropland, with a decrease in trees and bushes; recent developments are noted with a tinge of regret - Decrease in frogs since the change in the irrigation systems, as canals are no longer needed/filled with water
FLE	- Change of farm types in the landscape (in terms of both strategy and size) - Agricultural machinery seen as the reason for a field size increase and a reduction in hedgerows and trees - Windmills as a conflict point, due to perceived unfair farm allocation - Growth of a nearby city, including a larger road, a trainline and more traffic
QUP	- Socialist industrialization in the 1960s had a huge impact on the landscape by making small strip-like fields huge; perceived as something for which today’s farmers are not responsible - Decrease in field size after German reunification - Private efforts to plant hedgerows as wind protection and biodiversity promotion areas in a part of the study area - Infrastructure projects after reunification (e.g. highway, settlements) changed the landscape; often linked to the resulting loss of agricultural land, i.e. a loss of instrumental landscape value
IEV	- Cutting down apple trees and hedgerows was a central topic - Decrease in trees/hedgerows linked to subsidies given to cut them down in the early 1960s, but also to the farmers’ desire to modernize the farm - Development considered positive, with single remarks of regret about fragmentation of the ‘bocage’ (hedgerow system)
REU	- Land consolidation and melioration in the 1980s triggered a dynamic period of landscape change, with fields becoming larger and wetlands being drained and turned into farmland - Part of the land consolidation/melioration effort was the addition of hedgerows and the creation of a new nature reserve - Reception of the nature reserve ranged from annoyance, as it meant land could not be used for agriculture, to praise for its value in promoting biodiversity and as a recreational area - Decrease in single trees within meadows and fields (mostly fruit trees) due to cold winters, tree felling subsidies from the state, and to make fields more workable with machinery
TUR	- Abandonment of terraces and forest regrowth were central topics; linked to an increase in wild animals - Melancholy toward development; view that younger people no longer value home-grown food - Intensive grassland introduced during socialism leading to loss in meadow diversity

^1^See Table 1 for full names of study sites

### Comparison of findings from the two methods

In the following, the results of the two methods are compared and summarized.

In SMP, there is evidence from both methods of an increase in field size as a marker of agricultural intensification, especially from 2002 to 2017. This links well to the development and simultaneous land consolidation mentioned in the OHIs, as well as the drive for further specialization on arable land. What is only evident in the OHIs is that there were further consequences of this development on landscape structures and flora and fauna.

In FLE, both methods show the expansion of the local town Dronten, but few interviewees talked about it, which makes the mapping useful to underline this development. The OHIs, on the other hand, show the relevant connection between the development of civil infrastructure and increased activity in the landscape to perceived landscape change. While an increase in field size is difficult to gauge here, insights from the OHIs can explain why this change was difficult for the algorithms to classify: as FLE was constructed for agriculture, a certain standard plot size was defined, which was initially used for multiple crops but was reduced over time, ultimately suiting only one or two crops. While the algorithm was successful in differentiating the plots, the seemingly smooth transition from one crop to another in the 1981 aerial image made it difficult to identify the different crops in the field. This very specific differentiation between plot size and the area on which one crop is planted was a topic in the OHIs, with interviewees explaining that their fields did not increase in size, but rather the amount of crops per field decreased. Although not captured due to the generalized land-cover categories, windmills were quite impressively segmented in the 2000 and 2020 images, and they were also a major theme in the OHIs.

The mapped increase in field size in QUP between 1949 and 1975 overlaps with the accounts from the OHIs of the industrialization phase of the planned economy during socialism. The subsequent decrease in field size is explained in the OHIs with the reorganization of agriculture after the collapse of socialism, but it would have been expected to have already started between 1986 and 2006, which may be an indication that the mapped field sizes for 2006 have some uncertainties. Although not fully identifiable, due to the coarse land-cover categories and to misclassifications, the appearance of windmills and a highway and their timing coincide with the OHI statements. While these changes appear to occupy a small area compared with the whole study area, the OHIs provided the additional personal insight that these changes had quite an impact on both the visual appearance of the landscape and the instrumental value, as this land could no longer be used for agriculture. The planting of the windbreak hedgerows mentioned in the OHIs was not classified clearly in the land-cover maps.

In IEV, both methods show an increase in field size, with the mapping results showing a continuous increase. The OHIs closely linked the increase in field size to the decrease in trees and hedgerows, but this could not be distilled in the mapping, due to the very general categories used and the poor delineation of hedgerows and trees. The increase in arable land is in line with the agricultural development mentioned in the overall OHIs, with interviewees often focusing mainly on grassland at the beginning of their careers and increasing crop production since then.

The impact of land consolidation/melioration mentioned for REU in the OHIs is also reflected in the aerial imagery, as the change from a predominantly grassland/wetland area and small crop fields to a simplified landscape structured by fields and increased cropland. Another factor contributing to this simplification is the decrease in trees, both mentioned in the OHIs and visible as a decrease in small forest/tree geometries within the grassland between 1949 and 1980.

The OHIs from TUR indicated a clear trend of abandonment and forest regrowth, which is closely linked to a sense of grief. Over the study period, parts of the forest appear to have grown in the land-cover maps and aerial images. However, there are also massive clear-cuts visible in the spatial data between 2003 and 2018, which were not recorded in any of the OHIs. According to the OHIs, the change toward intensification of grassland and away from crop production already started during socialism.

In conclusion, both methods allowed insights into characteristic landscape changes for the study sites (Table 5). Thanks to the ability to distinguish between cropland and further arable land, a change in field size could be shown from the aerial imagery. Apart from using field size change as a proxy for management changes, the landscape mapping was limited to the land-cover aspect of landscape. The OHIs, on the other hand, pointed to a wide array of landscape changes, with physical landscape changes often referred to through changes in farm management and land use. In many study sites, the increase in the size of arable fields was widely discussed in the OHIs, which is also reflected in the landscape mapping (Fig. 3). The OHIs provided evidence of landscape changes that could not be mapped due to their size (i.e., spatial resolution not sufficient) or thematic detail (e.g., wind turbines, ditches, single trees, or linear green elements). Further, some landscape changes identified as important in OHIs (e.g., the building of new highways and train lines in QUP, which reduced agricultural land) only covered a small part of the area covered by the aerial image, which has to be considered in comparisons based on the spatial extent of land-cover classes. An interesting result was achieved for TUR, where mapping revealed that large forest patches were clear-cut between 2003 and 2017. However, none of the interviewees mentioned this, and they only reported forest expansion into sloping and remote agricultural land. Further, the OHIs provided ample information on drivers behind observed landscape changes, leading to a better understanding of the processes.

**Table 5:** Contributions of aerial images, oral history interviews, and overlaps between the two methods for studies of long-term landscape change

Aerial images	Overlap	Oral history interviews
- Quantification of land-cover changes - Outsider’s perspective/algorithms - Localization of land-cover changes^1^ - Estimation of field size as a landscape-level indicator for agricultural intensity change - Multiple time steps illustrate/date the frequency/dynamic of change	- Observations of LULC changes - Changes in landscape structures, e.g., size of arable fields; landscape elements like hedges or single trees^a^	- Focus on farm changes related to management - Relevance of landscape change to visual perception and daily life, independent of actual magnitude - Landscape changes described in connection with their drivers - Landscape changes associated with instrumental and relational values

^1^Potentially possible based on aerial images, not attempted in this analysis

## Discussion

### Methodological considerations

In this study we found a strong complementarity between two fundamentally different approaches to analyzing historical landscape change. Each of the individual methods has limitations and inaccuracies, but together they lead to better insight into the landscape dynamics. Comparison can indicate (dis)agreement between mapped landscape change based on remote sensing and landscape change as remembered by local stakeholders. This provided, for example, insights into landscape changes that could not be mapped, or land-cover changes that were only mapped for small areas but were remembered as having a large impact.

While our remote sensing analysis was able to facilitate land-cover change mapping at a high speed, allowing for image repetition in several time steps and multiple study landscapes, we also encountered several limitations of the standardized workflow. Our model accuracies based on test data were comparable to those in other studies using BW aerial imagery in which multiple land-cover classes were classified (Adugna et al. 2022; Kindermann et al. 2023), but they were lower than in studies focusing on only one land-cover class (Vogels et al. 2017; Whiteside et al. 2020). Applying the model on the segmented polygons brought together the uncertainties of the segmentation and the classification. Uncertainties were introduced through too coarse/fine segmentation, as well as misclassifications due to similar gray shades for different land-cover classes or due to polygons covering more than one land-cover type. In the arable landscape classified using MA, cropland was classified relatively well, while other land-cover classes were occasionally also classified as cropland. For the mixed landscape classified using MM, grassland—cropland and grassland—forest confusions were often observed. Since historical aerial BW imagery has limited discriminative power, as it has only one intensity channel to begin with, mixing different image qualities (i.e., for different study sites and time steps) added another challenge, due to varying image noise and spatial resolution. In addition, depending on the type of landscape, the sensor, the phenological season, and the lighting conditions, the same land-cover feature can be described by different spectral, textural and geometric properties. To balance the reliance on one spectral band, we applied texture metrics/geometry, which improved the analysis but was limited by the aforementioned challenges and by segmented geometries that were not always reliable. To address these issues, context-aware machine learning may be a way to increase the predictive power of land-cover classification based on BW aerial imagery (Ratajczak et al. 2019, Khan and Bassalamah 2023). Another way to improve the reliability of the results would be to train models specifically based on land-cover categories, variables and training data that suit the local conditions (Kindermann et al. 2023). A further option could be a post-processing of the result based on logical rules in a geographic information system (e.g., no small polygons of the built-up category allowed within cropland) or manual corrections. While there are still issues with the automated land-cover classification of BW imagery, we see an automated approach as a step toward the possibility of classifying a variety of landscapes over larger regions and more time steps than manual mapping would allow (Ratajczak et al. 2019). More time steps mean a higher temporal resolution and thus a better dating of landscape change processes and an improved ability to highlight more or less dynamic times, which can be an asset for e.g. landscape connectivity studies (Uroy et al. 2021).

Mapping change in several time steps also makes it possible to more accurately date and spatially allocate information from OHIs. Even though possibilities of remote sensing increase with higher spatial and temporal resolutions, as well as higher computing power, what is mappable is limited to what can be ‘seen’ from above. This can lead to the misinterpretation of change, but also might not capture full processes, e.g., excluding parts that take place below the canopy (Fox et al. 2017). In addition, when establishing a land-cover classification, decisions made by the scientist about research questions, as well as what should be mapped and how, influence the processes that are captured (Messerli et al. 2009; del Río-Mena et al. 2023).

The OHIs proved to be a valuable tool for gaining insight into landscape change over several decades, which is consistent with findings from other studies (Bürgi et al. 2017; Nimmo et al. 2020; Zhou et al. 2022). Often, landscape changes were mentioned in ‘logical’ packages, such as the interlinkage between field size increase and the simultaneous decrease in the number of trees. Further, many interviewees mentioned drivers associated with some of the changes. These drivers were often connected to an event or period, such as the land consolidation in REU, the further development of the irrigation system in SMP, or the industrialization phase during socialism in QUP. Finally, positive and negative connotations were attached to the observed landscape changes, typically when the landscape change triggered an alignment or distortion with instrumental or relational landscape values. For farmers in IEV, for example, the expansion of farming was both an opportunity to rationalize and mechanize farm work and a sign of progress.

While we only considered changes during the lifetime of the interviewees, which more or less coincided with the aerial imagery, there are some studies that use participatory approaches to reconstruct landscape change further back in time than spatial data are available, relying on oral tradition (Brown et al. 2018). As we asked about landscape change in general during the OHIs, a variety of aspects were mentioned, including attributed landscape values and drivers of change. Depending on the focus of the study, it could be worthwhile to more specifically ask about relational and instrumental values attributed to landscape changes to gain an even deeper insight in the meaning of landscape change for the local population (Stenseke 2018; Riechers et al. 2022; van Noordwijk et al. 2023) and its impact on their wellbeing (Fagerholm et al. 2016). We considered only farmers for the OHIs, which evidently impacted the narratives of landscape change; responses could vary across stakeholder groups (Ujházy et al. 2020; Frei et al. 2022). Depending on the aim of the study, diversifying the demographic and professional backgrounds of the interviewees would provide a broader local perspective on landscape change and interesting insights into diverging views. Moreover, when interviewees are asked about landscape change in a normal interview setting, it is difficult to place the mentioned change in the landscape at a later point. For this challenge, participatory mapping approaches are an important research avenue (Fagerholm et al. 2022). Letting interviewees map the location where they perceived landscape changes also could help in assessing whether differing narratives of interviewees on landscape change—as sometimes encountered in our interviews – are based on different subjective perspectives or result from interviewees referring to different parts of the landscape. When analyzing causes/driving forces of landscape change, it is beneficial to combine OHIs with complementary source types, such as newspaper articles, planning documents or local statistical data (Kerselaers 2013, Eiter et al. 2016), in order to compensate for the limitations of individual sources (Cresswell 2003) and to account for different discourses of historical development (Bürgi et al. 2013; Abrams 2016).

### Challenges and opportunities for mixed-method approaches

Mixed-method approaches for identifying and better understanding landscape changes face several challenges and opportunities, both thematic and methodological. However, the combination of remote sensing and qualitative stakeholder information on landscape change provides two essential perspectives for a better understanding of ecosystem services and human well-being in changing landscapes. When comparing these two sources, it should be kept in mind that both methods have specific strengths and qualities in bringing insights into landscape change and results should be interpreted accordingly. For example, if interview statements deviate from ground-truthed mapped landscape processes, they are only wrong and unreliable regarding the mapping aim. However, they still provide invaluable information about the understanding of landscape change by providing insight into stakeholders’ landscape perceptions and how they are remembered and reported (Abrams 2016). In such a case, following up on the reason for the mismatch could enrich the analysis greatly through fostering an understanding of different perspectives on landscape change (Knierim et al. 2021). For example, the case observed in TUR fits into this pattern. While it is very clear from the remote sensing image that there are clear-cuts, it is not in the farmers’ narrative of landscape change. An interpretation of this discrepancy is that clear-cuts are perceived as a result of standard forest management practice/disturbance (Senf and Seidl 2021) and therefore not remembered as a landscape change.

When bringing qualitative and quantitative data together, the order in which the approaches are used is essential. In this study, data for both methods was collected and analyzed individually and then triangulated. Addressing stakeholders first has the advantage that observations that were important to the interviewees or important information about certain management changes can help to inform decisions on which landscape changes to map (Isager and Broge 2007; Berget et al. 2021). Further, it can give insights into temporal land uses (Mathur and Bhattacharya 2023) or land uses that are not captured through remote sensing data (Fox et al. 2017). An exchange with local stakeholders further makes it possible for landscape changes seen as important by the stakeholder to be prioritized by the remote sensing approach, such as the change in number of trees and hedges in IEV, REU and SMP. Also, local knowledge through the stakeholder perspective can point to land-use practices that are hard to recognize from ‘above’ or improve the understanding, and hence the mapping, of dynamic systems like small-scale swidden agriculture (Isager and Broge 2007; Fox et al. 2017; Berget et al. 2021).

Mapping landscape changes before collecting the local stakeholders’ perspectives, on the other hand, has the advantage that hot spots of LULC can be detected inductively, and interviewees can then be approached about why changes occurred in certain places (Dimopoulos and Kizos 2020). Of course, the two approaches could also be combined by, e.g., conducting a first series of interviews to support the mapping procedure, followed by a second series in which the results of the mapping are discussed with local stakeholders. However, such combinations are time intensive. In any way, a mixed-methods approach is a step toward transdisciplinary research efforts, which are important for building momentum for transformative change based on local solutions.

## Conclusions

Mixed-methods approaches that combine information on physical land-cover change with local stakeholders’ perspectives make it possible to capture widely different dimensions of landscape change. Combining these approaches must be done based on insights into the strengths and limitations of both approaches. There are, for example, still caveats in the automatic classification of historical aerial imagery and the reliable identification of land-change processes. OHIs lack the power to quantitatively report on landscape change, but they provide invaluable information about the local perception and impact of landscape change and its drivers. The combination of top–down remote sensing information and bottom–up local insights not only offers a gain in content, but also can trigger exchange between two different ways to approach landscape change. Bringing the local perspective closer to scientists and policy makers and a broader landscape perspective to the stakeholders could be an important cornerstone in efforts to design targeted landscape-level solutions for more sustainable landscape development.

### Supplementary Information

Below is the link to the electronic supplementary material.

Supplementary file1 (PDF 424 KB)

Supplementary file2 (PDF 223 KB)
