# Supplementary material for: Exploring agricultural landscape change from the second half of the twentieth century onwards: combining aerial imagery with farmer perspectives
Source: Landsc Ecol. 2024 Jun 20;39(7):120. doi: 10.1007/s10980-024-01914-z (PMC11189988; doi:10.1007/s10980-024-01914-z)
Supplement: Supplementary file 1 — Supplementary file1 (PDF 424 KB) [file 10980_2024_1914_MOESM1_ESM.pdf]

Exploring agricultural landscape change from the second half of the 20th century onwards: Combining aerial imagery with farmer perspectives. Mohr F, Pazur R, Debonne N, Dossche R, Helfenstein J, Hepner S, Levers C, Verburg PH, Bürgi M.
Submitted to Landscape Ecology.

Supplementary information I:

Additional information remote sensing

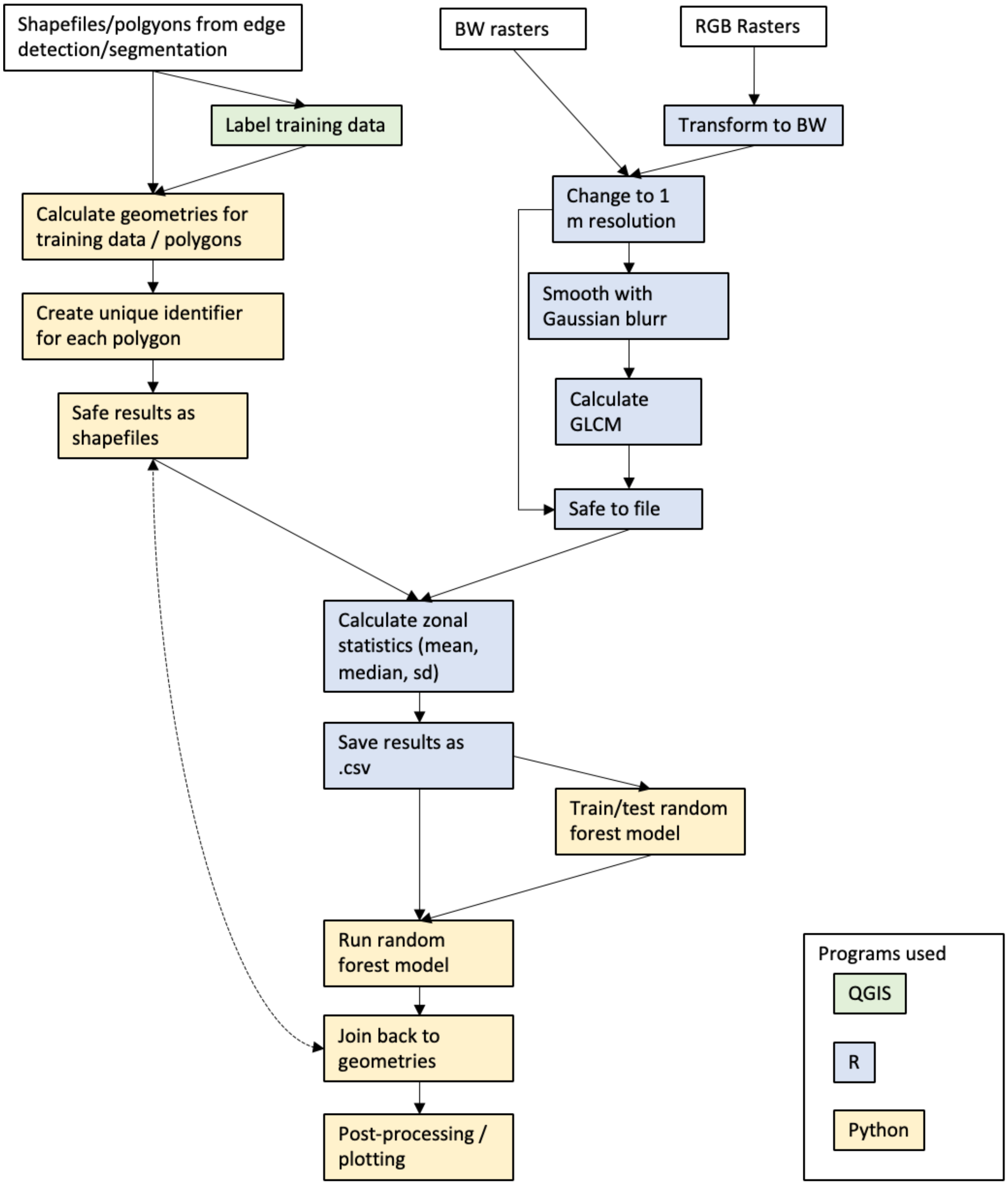

Fig. 1 Schematic visualization of the classification approach and the programs used

Table 1 List of predictor variables used for random forest classification

Type	Basis	Measure	Literature examples
Gray intensity	BW raster (RGB transformed previously); both resampled to 1m and smoothed with Gaussian Blur.	Zonal statistics - Median & Standard deviation	Kindermann et al. 2023, Vogels et al. 2017
Geometry	Polygons resulting from Segmentation	Area	Kindermann et al. 2023, Vogels et al. 2017
		Shape index / Iso-Perimetric Quotient $\frac{4\pi * area}{perimeter^2}$	Hernandez-Suarez et al. (2022),
Texture (input = raster described in row gray intensity)	GLCM Contrast	Zonal statistics - Median	Hall-Beyer M (2017), Vogels et al. 2017
	GLCM Correlation	Zonal statistics - Median & Standard deviation	Hall-Beyer M (2017), Vogels et al. 2017
	GLCM Entropy	Zonal statistics - Median & Standard deviation	Hall-Beyer M (2017), Vogels et al. 2017

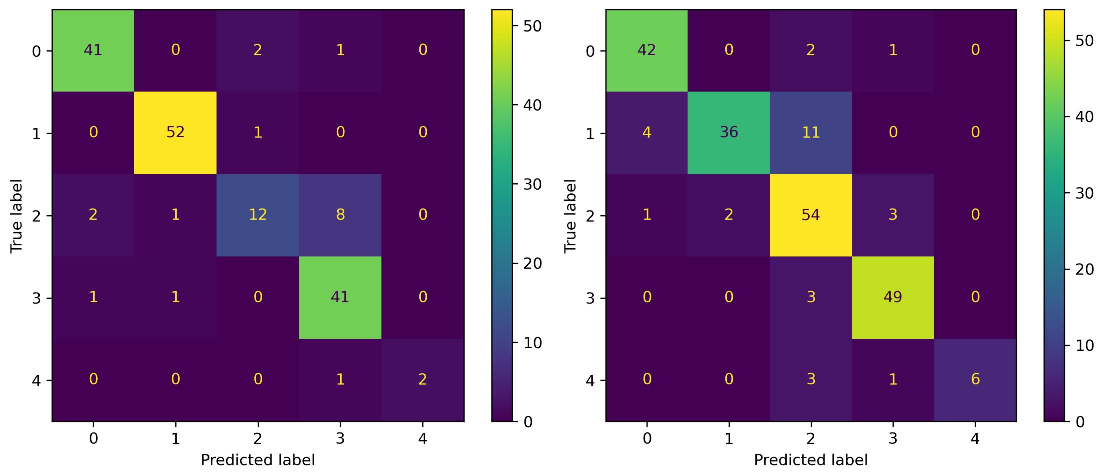

Fig. 2 Confusion matrix for the model trained on the labeled data from arable landscapes (SMP, FLE, QUP; left) and mixed landscapes (IEV, REU, TUR; right). 0 = built-up, 1 = cropland, 2 = grassland, 3 = forest/trees, 4 = water. Numbers in the confusion matrix indicate the number of test polygons.

Table 2 List of corrected values for Fig. 6 in the manuscript. 1 = built-up, 2 = cropland, 3 = grassland, 4 = forest/trees, 5 = water, 9 = N/A. Changes from grassland to cropland to grassland and vice versa were not corrected for.

SMP, FLE, TUR		REU		QUP, IEV	
original	corrected	original	corrected	original	corrected
121	111	1211	1111	12111	11111
131	111	1311	1111	13111	11111
141	111	1411	1111	14111	11111
151	111	1511	1111	15111	11111
212	222	1121	1111	11211	11111
242	222	1131	1111	11311	11111
252	222	1141	1111	11411	11111
313	333	1151	1111	11511	11111
343	333	2122	2222	11121	11111
353	333	2422	2222	11131	11111
414	444	2522	2222	11141	11111
424	444	2212	2222	11151	11111
434	444	2242	2222	21222	22222
454	444	2252	2222	24222	22222
515	555	3133	3333	25222	22222
525	555	3433	3333	22122	22222
535	555	3533	3333	22422	22222
545	555	3313	3333	22522	22222
		3343	3333	22212	22222
		3353	3333	22242	22222
		4144	4444	22252	22222
		4244	4444	31333	33333
		4344	4444	34333	33333
		4544	4444	35333	33333
		4414	4444	33133	33333
		4424	4444	33433	33333
		4434	4444	33533	33333
		4454	4444	33313	33333
		5155	5555	33343	33333
		5255	5555	33353	33333
		5355	5555	41444	44444
		5455	5555	42444	44444
		5515	5555	43444	44444
		5525	5555	45444	44444
		5535	5555	44144	44444
		5545	5555	44244	44444
				44344	44444
				44544	44444
				44414	44444
				44424	44444
				44434	44444
				44454	44444

References:

Hall-Beyer M (2017) Practical guidelines for choosing GLCM textures to use in landscape classifica-tion tasks over a range of moderate spatial scales. Int J Remote Sens 38(5):1312–1338. https://doi.org/10.1080/01431161.2016.1278314

Hernandez-Suarez JS, Nejadhashemi AP, Ferriby H, Moore N, Belton B, Haque MM (2022) Performance of Sentinel-1 and 2 imagery in detecting aquaculture waterbodies in Bangladesh. Environ Model 157:105534. https://doi.org/10.1016/j.envsoft.2022.105534

Kindermann E, Hölzel N and Wellstein C (2023) Combining historical aerial photography with machine learning to map landscape change impacts on dry grasslands in the Central Alps. Landsc Ecol 38(8):2121–2143. https://doi.org/10.1007/s10980-023-01684-0

Vogels MFA, de Jong SM, Sterk G, Addink EA (2017) Agricultural cropland mapping using black-and-white aerial photography, Object-Based Image Analysis and Random Forests. Int J Appl Earth Obs Geoinf 54:114–123. https://doi.org/10.1016/j.jag.2016.09.003
