# Supplementary material for: Exploring agricultural landscape change from the second half of the twentieth century onwards: combining aerial imagery with farmer perspectives
Source: Landsc Ecol. 2024 Jun 20;39(7):120. doi: 10.1007/s10980-024-01914-z (PMC11189988; doi:10.1007/s10980-024-01914-z)
Supplement: Supplementary file 2 — Supplementary file2 (PDF 223 KB) [file 10980_2024_1914_MOESM2_ESM.pdf]

Exploring agricultural landscape change from the second half of the 20th century onwards: Combining aerial imagery with farmer perspectives. Mohr F, Pazur R, Debonne N, Dossche R, Helfenstein J, Hepner S, Levers C, Verburg PH, Bürgi M. Submitted to Landscape Ecology.

Supplementary information II:

Additional information oral history interviews

Table 1 Overview of identified categories and themes related to landscape change from the oral history interviews.

Theme	Category
farm management	additional barns/siloes/modernization
	irrigation system
	field size (increase)
	field size (decrease)
	canals/ditches for irrigation/drainage (change)
	more tulips fields
	clean field / field edges
	increase in organic farming
	greener field margins & farm level nature projects
	change composition of farms
	abandonment of fields/farms
	less sheep
	leveling land / destroying terrace
	interrelationship soil/management
	focus solely on crops/arable
landscape structure	vegetation (increase)
	lagoons/wells (decrease)
	trees (decrease)
	trees (increase)
	linear green elements (decrease)
	linear green elements (stable)
	linear green elements (increase)
infrastructure	waste water collection
	new highway / trainline
	nature conservation
	more wind turbines
	bigger settlement / construction
	field path / roads (decrease)
	field paths / roads (increase)

	more recreation areas
fauna / flora	animal (decrease)
	animals (increase)
	plants (decrease)
	plants (increase)
land cover	field to forest
	mainly arable land (stable)
	fruit plantation to arable land
	wetland/grassland to arable
no change	-
level of activity	sound of windmills
	increase in population / don't know people anymore
	more agricultural traffic
	busier
	local engagement for landscape and community

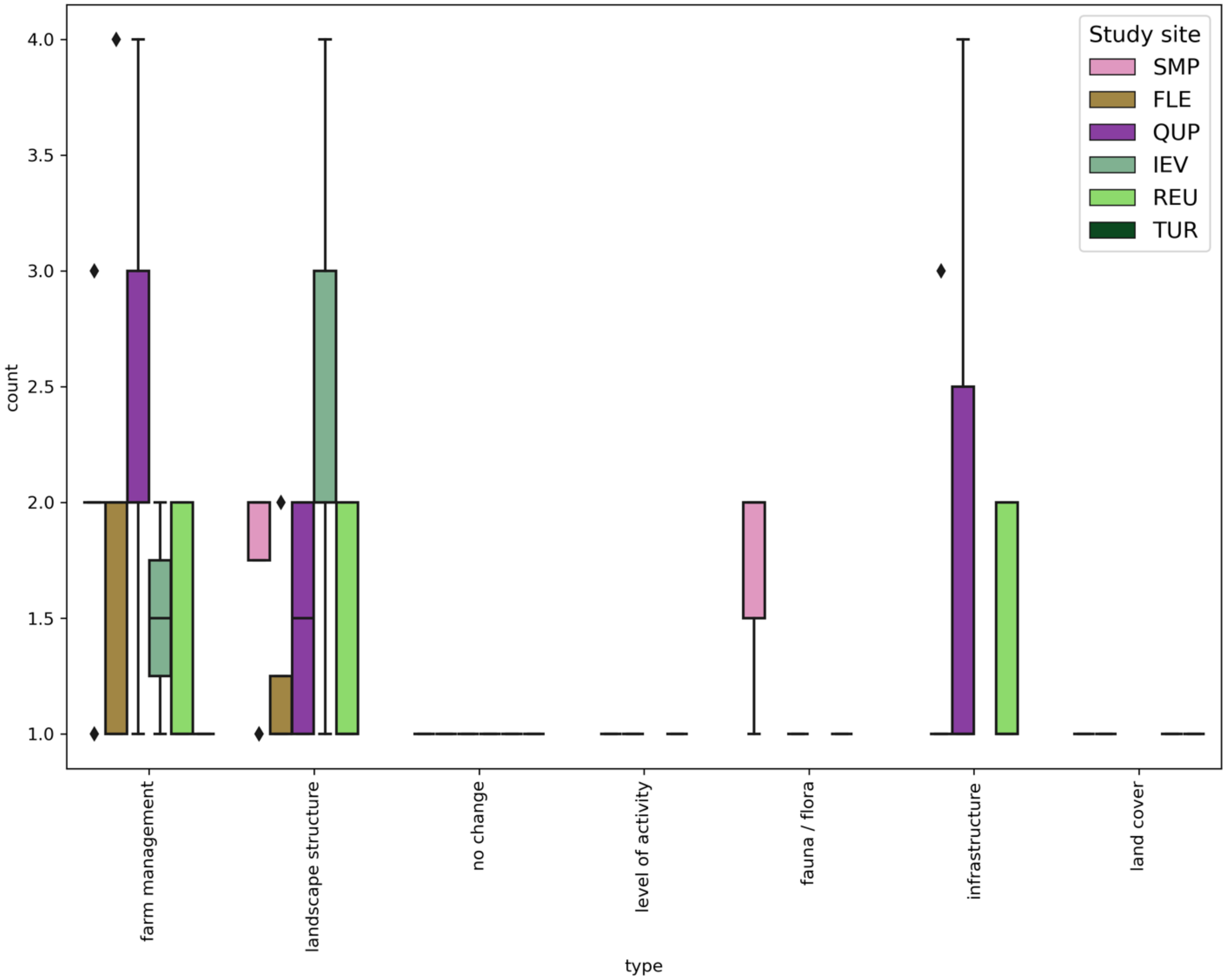

Figure 1 Boxplot showing the diversity of how many landscape change categories within a landscape change type were mentioned by farmers in the different study sites. The box indicates the first and third quartiles, the line indicates the median, the whiskers indicate the minimum and maximum, and the diamond indicates the outliers.

Narratives of landscape change based on oral history interviews

The following are narratives of landscape change based on the oral history interviews for each study site, written to a) summarize all coded landscape changes (Fig. 6 in the manuscript) and b) integrate typical drivers and landscape values touched upon in describing/remembering landscape change. These narratives provide the background for Table 4 in the manuscript. See Table 1 in the manuscript for full names of study sites.

SMP

In SMP, all of the observations made regarding landscape change were related to agriculture. The combination of landscape changes, such as the increased dominance of cropland and the leveling of land, is a strong reflection of agricultural intensification at the landscape level. This development was further enhanced through the introduction of a large-scale irrigation infrastructure in the 1960's and a renewal thereof in the 2000's, which both times went together with a land consolidation, enabling the farmers to increase their field sizes. The introduction of the irrigation system had an impact on the perception of the landscape as such, e.g. through visible irrigation infrastructure or a change in vegetation due to increased humidity. Through the renewal of the irrigation system, open irrigation canals were replaced with underground pipes. While poplars were planted along the irrigation canals with the development of the first irrigation system, the number of trees, shrubs and wells has decreased in recent years, both because of the space needed for land consolidation and to prevent roots from destroying the newly installed underground pipes. One farmer does not understand this development as he thinks it would still be possible to plant mulberry trees or shrubs - but he also points out that most farmers like to have clear areas without things to get in their way, so everything was burned. All farmers report a decline in frogs, birds (especially linnets) and (beneficial) insects. While some attribute this to the use of herbicides, others attribute it to the lack of water in the irrigation ditches since the switch to underground pipes, which some interviewees find unfortunate: "For animals, the same [decrease]. For example, partridges, I see them looking for water near my sheepfold, but there is no water anywhere (SMP F10)". Pest insects, on the other hand, have increased according to one farmer. The decrease in frogs was also attributed to the so-called "frogmen" who caught frogs in the 1980s/90s and sold them to restaurants.

FLE

When the FLE was designed, it was intended to be used for farmland, with predefined plots that would be used to grow a variety of crops. Since then, interviewees have observed an increase in livestock farms and a decrease in fruit farms. Rather than describing an increase in field size, the discourse was about a reduction in the number of crops per plot (which is effectively an increase in the area used per crop). Furthermore, many changes in landscape-level management were associated with an increase in the size of barns, machinery, and even farms themselves (up to 400-500 ha on the polder). More agricultural traffic was also mentioned. The larger agricultural machinery was seen as a reason for the increase in field size, as well as the reduction of some hedges and trees. The latter also reportedly created a more open landscape. On the other hand, an interviewee who was one of the first farmers also welcomed the general increase in vegetation since the creation of the Flevopolder. Another interviewee noted the greener field

margins and their positive effect on wasp reduction. He also praised the organic farms for making the polder a brighter place. When it comes to landscape changes related to infrastructure, the appearance of windmills is a prominent feature. In this study area, the government defined areas based on lines where farm owners could build windmills. Because not all farmers were able to take advantage of this economic opportunity, interviewees linked this landscape change to deep frictions among the local population and a decrease in social cohesion. Some interviewees who happened to be among those who were not able to build windmills thought they were ugly or were bothered by the noise. Other interviewees reported that the landscape had become more crowded due to railroad tracks, a larger road, and more recreational areas associated with the growth of the nearby town of Dronten. While some interviewees did not perceive any changes in biodiversity, others remembered a sharp decline in woodpeckers, pheasants and rabbits/hares, especially in the first decade after the start of cultivation (late 1970s). One interviewee believes that the greening of field margins is a hiding place for mice and therefore does not do it for certain crops. Interviewees reported more magpies, foxes and rats (even in the villages) and blamed these animals for the decline of smaller animals, e.g. foxes disturbing bird nests. Since the 1980s, frogs have returned to the area, whereas before they could not survive there. Some interviewees were also pleased to see the return of (bird) species such as kingfishers, larks, swallows and owls. Increases in biodiversity are generally attributed to an increase in organic farms and greening, as well as a decrease of pesticides. However, a farmer points out the need for policy to keep up greening development and the maintenance thereof.

QUP

In QUP the biggest changes in landscape were attributed to the industrialization phase of agriculture starting in the late 1960s/70s that transformed the landscape by enlarging the fields up to 200 ha and reducing field paths and other structures. This change was sometimes even referred to as something that the last generation of farmers / GDR leaders were responsible for and not the current farmers. The increase in field size was - next to the “industrialization” discourse in the GDR at the time also explained as a need since also “in the east” technology advanced, and bigger fields were needed to make it worthwhile to have larger technology. The large fields however increased rain and wind and rain erosion. One interviewee - fresh from his studies - started to plant hedges as wind protection and biodiversity enhancement measure in 1981. The farm where he was formerly working still keeps this up. A decrease of field (still field of 60, 80 ha!) was remembered after the reunification of Germany in 1990, as well as the re-construction of field paths, due to an increase in farms. However, there is also a decrease in crop rotation noted as many farmers tend to focus on crops with a good market price. The field sizes are also often cited as a reason for the decrease/disappearance of small game like hare, hamsters, partridges and pheasants from the GDR onwards. Further a decrease in insects was noted by some farmers. A common narrative links the decline of insects to the decline of dung heaps in the landscape/farms and drier weather. Lack of nesting sites is also cited as a reason for the decline of birds, or crows as predators of smaller birds. It becomes clear that certain animals are more popular than others (e.g. crows, wild boars). Some farmers recalled that in the post-war years there were much more elements of subsistence agriculture in the landscape, such as the use of roadside ditches and railroad embankments to plant cherry trees, gooseberries, or currants, or to cut grass to feed goats and rabbits. These elements have since been lost, and some interviewees noted that roadsides and field margins are usually mowed too early, which -

unfortunately - reduces the opportunity to create space for biodiversity. However there are also farmers that pointed out private initiatives next to obligatory greening measures: “We are now more and more interested in replanting bushes or areas that are difficult to cultivate, so that we can create, let's say, a kind of insect pasture or something. [...] That's three hectares that are not cultivated here, or that are difficult to cultivate, and then you do things like that. [...] But we do it anyway, because we say, what do we want to do with our big machines in such corners, we can rather do something for nature” (QUP F5). While farmers stated that there are things that could be improved in terms of biodiversity, they also noted a big improvement since the GDR, such as the increase in songbirds and structural elements: “Even if people now always say: "Yes, the huge agricultural areas, the biodiversity has become much worse." They should have come before” (QUP F9). Since the reunification, there was further building activity for highways/roads, train lines, settlements, industrial areas and wind turbines, which was all attributed as land “lost” for agriculture and impacting the visual landscape (for more details see Mohr et al. (submitted)).

IEV

In IEV many interviewees, changes in landscape structure were mentioned prominently when asked about landscape change, especially cutting down of apple trees and embankments/hedges. This development is closely linked to the increase in field size (farm management change). Often these landscape changes were accompanied by a narrative (and pride) of wanting to modernize agriculture along the prominent discourse by the state/unions at the time: “To modernize, you couldn't stay with small plots of land everywhere. They started by cutting down the apple trees, by giving subsidies to cut down the acid apples in the years 1962 and 1963. We received incentives for cutting down apple trees” (IEV F2). Most farmers seem to be happy with the development, as it has made farming easier and more feasible; one farmer even praises the areas that have changed as progressive, while calling those who have not taken the opportunity to increase the size of their fields as backward and having missed the opportunity, as this is no longer possible due to current legislation. Some of the interviewees were also concerned about the fragmentation of the "bocage" and spoke of private projects to either save some hedges or plant a small forest for the next generation. While one interviewee did not see any changes in biodiversity, others noticed the decrease/disappearance of birds, snakes and animals in general. A farmer attributes this to abuses of i.e. herbicides, but in the same sentence underlines that farmers are the “first ecologists”. Others observe an increase in biodiversity since the modernization phase (until 1980’s), because farmers are more careful with pesticides than they used to be.

REU

In the 1980s, REU experienced a large-scale land consolidation and melioration, which was seen as a major intervention in the landscape. At the heart of this development was the transformation of what had previously been predominantly wetland/grassland in the valley floor into arable land through drainage and melioration. New roads, drainage canals, and hedgerows (a mandatory requirement) were added, while land consolidation made it possible to increase the size of fields. At the same time, part of the Reuss valley was declared a nature conservation area. While some farmers were annoyed at the loss of

agricultural land to nature conservation ("Exactly that land was the best agricultural land"), others appreciated the new element in the landscape and talked about using it as a recreational area or as a gain for nature:: " I don't remember wisdom sage, for example, in my childhood. Things like that. In the past it was certainly an intensively used agricultural landscape, and today you have these windows again, where there is room for ecology and where it is actively promoted" (REU F9). In general, farmers perceive plant diversity to be stable or to have increased as a result of ecological interventions, with one interviewee even appreciating that new ecological flower meadows have changed the appearance of the landscape. However, interviewees noted a decline in insects, birds (swallows, pheasants) and fish. One farmer remarks that in the past there was more time to e.g. protect bird nests, but in today's agriculture everything is timed and programmed. In recent years there has been an increase in beavers, which was not appreciated as they fell trees important for flood protection from the local river. Throughout the study period, there was a large decline in fruit trees. While a government intervention in 1950 to reduce fruit production for liquor production led to the cutting of trees, some trees also died during cold winters (e.g. in 1963). Since then, increasing mechanization and the decreasing economic value of the high-stem fruit trees have made it more worthwhile to cut them down to make the land easier to manage. Whereas in the beginning of the study area there were mainly dairy farms, there are now also suckler cow farms or vegetable farms, with many farms applying voluntary, government-funded biodiversity measures. Some farmers noted a structural change towards fewer but larger farms.

TUR

While a variety of landscape change types were mentioned in most of the study sites, interviewees in TUR focused mainly on landscape change due to changes in farm management and associated changes in land cover. TUR is described as spotting mainly "classical pastures or meadows". During the socialist period, the trend started that more remote land was abandoned because it was not possible to mechanize work there. This trend continued and intensified for two reasons: During socialism, a lot of people would keep a small farm next to their day time factory job for subsistence and making some extra money. However, as this generation is getting older, there is hardly anyone who has an interest to take over the land for agriculture apart from the occasional use as vacation homes. This leads - to the resentment of some interviewees - to 'bad development', as the carefully tended land, meadows and terraces of their ancestors are overgrown and turned into forest. Related to this, respondents report a shift in value away from investing labor and time to produce good quality food for themselves: "And no one keeps animals anymore, no one works the land. We are used to homemade eggs for example, the ones I bought I don't even like the smell. After all, you only know what you are eating when you produce it yourself." Further, after socialism not all land was reclaimed because it was not attractive anymore to former owners or because regulations were unclear. In the flatter part of the study area, the confusing reclamation has had the opposite effect, facilitating illegal land grabbing after the collapse of socialism. There are also complaints that today's managers sometimes don't take good care of the land, for example by mulching their land instead of cultivating it properly. Some interviewees speak of a decrease in the diversity of meadow flowers, which some interviewees attribute to developments during socialism, such as mechanization, the introduction of "cultivated grasses" to increase yields, or pesticide spraying by

airplanes. While interviewees agree that there has been a large decrease in frogs and some bird species, not all interviewees agree on other changes: One farmer noted a decrease in insects/butterflies and snakes, while others clearly noted an increase in "bad" insects, snakes, voles and wild boars. The increase in snakes, voles, wild boar, or wildlife in general is generally attributed to the overgrowth of formerly cultivated land.
